# Closing the Fluid Gap: Improving Isotonic Maintenance Intravenous Fluid Use in a Community Hospital Network

**DOI:** 10.1097/pq9.0000000000000696

**Published:** 2023-10-07

**Authors:** Shraddha Mittal, Sheila Knerr, Julianne Prasto, Jessica Hunt, Carolyn Mattern, Tsae Chang, Ronald Marchese, Morgan Jessee, Lauren Marlowe, Josh Haupt

**Affiliations:** From the * Department of Pediatrics, Children’s Hospital of Philadelphia, Philadelphia, Pa.; † Department of Pharmacy, Virtua Voorhees, Voorhees, N.J.; ‡ Department of Pharmacy, Grand View Health, Sellersville, Pa.; § Department of Pharmacy, Penn Medicine Princeton Medical Center, Plainsboro, N.J.; ¶ Center for Healthcare Quality & Analytics (CHQA), Children’s Hospital of Philadelphia, Philadelphia, Pa.

## Abstract

**Introduction:**

The American Academy of Pediatrics recommends using isotonic intravenous fluids (IVF) for maintenance needs to decrease the risk of hyponatremia. We conducted a quality improvement project to increase the use of isotonic maintenance IVF in pediatric patients admitted to three sites in a community hospital network to >85% within 12 months.

**Methods:**

We used improvement methodology to identify causes of continued hypotonic fluid use, which involved provider behavior and systems factors. We implemented interventions to address these factors including: (1) education; (2) clinical decision support; and (3) stocking automated medication dispensing systems with isotonic IVF. We compared isotonic IVF use before and after interventions in all admitted patients aged 28 days to 18 years who received maintenance IVFs at the rate of at least 10 mL/hour. We excluded admissions of patients with active chronic medical conditions like diabetic ketoacidosis. Balancing measures were the occurrence of adverse events from hypo- or hypernatremia. Data were analyzed using Laney P′ statistical process control charts.

**Results:**

Isotonic IVF use among patients requiring maintenance fluids at all three sites surpassed the goal of >85% within 12 months. There were no reports of hypo- or hypernatremia or other adverse outcomes related to the use of isotonic IVF.

**Conclusion:**

A combination of interventions aimed at provider behavior and systems factors was critical to successfully adopting the American Academy of Pediatrics guideline regarding the use of maintenance isotonic IVF in hospitalized children.

## INTRODUCTION

Maintenance intravenous fluids (IVFs) are a common part of management for hospitalized pediatric patients.^1,2^ The basis for IVF management, established in the 1940–1950s, derived from Holliday and Segar’s article^3^ recommending weight-based fluid and glucose for maintenance. Subsequently, hypotonic maintenance IVF for pediatric patients became standard, although management varied widely.^3–5^ More recent data have shown an increased risk of hyponatremia when using hypotonic IVF as maintenance,^6–8^ and death and encephalopathy have been reported.^7,9^ In December 2018, the AAP released a new clinical practice guideline for pediatric fluid management recommending isotonic IVF for maintenance fluids for pediatric patients 28 days–18 years of age.^10^ This has been a significant shift in management for pediatric hospital medicine.^11,12^

Evaluation of isotonic IVF use at our three community hospitals showed that more than half of our admitted pediatric patients requiring maintenance fluids were still receiving hypotonic IVF 1 year after the publication of the American Academy of Pediatrics (AAP) guideline. We undertook a quality improvement (QI) project to increase the use of isotonic IVF for hospitalized pediatric patients across all three hospitals. Our goal was to increase the proportion of isotonic maintenance fluid orders in pediatric patients aged 28 days to 18 years to more than 85% within 12 months. This article was written according to the Standards for Quality Improvement Reporting Excellence 2.0 guidelines.^13^

## METHODS

### Context

The project setting involved three community hospitals across two states affiliated with a large, tertiary care pediatric healthcare system, which collaborate on QI projects. All sites follow evidence-based guidelines and clinical pathways from the tertiary care center. Pediatric hospitalists manage all patients on the inpatient units, a group of 22 hospitalists care for approximately 1,700 admissions at site 1, 10 hospitalists for 800 admissions at site 2, and 7 hospitalists care for 400 admissions at site 3 annually.

### Interventions

**Fig. 1.**
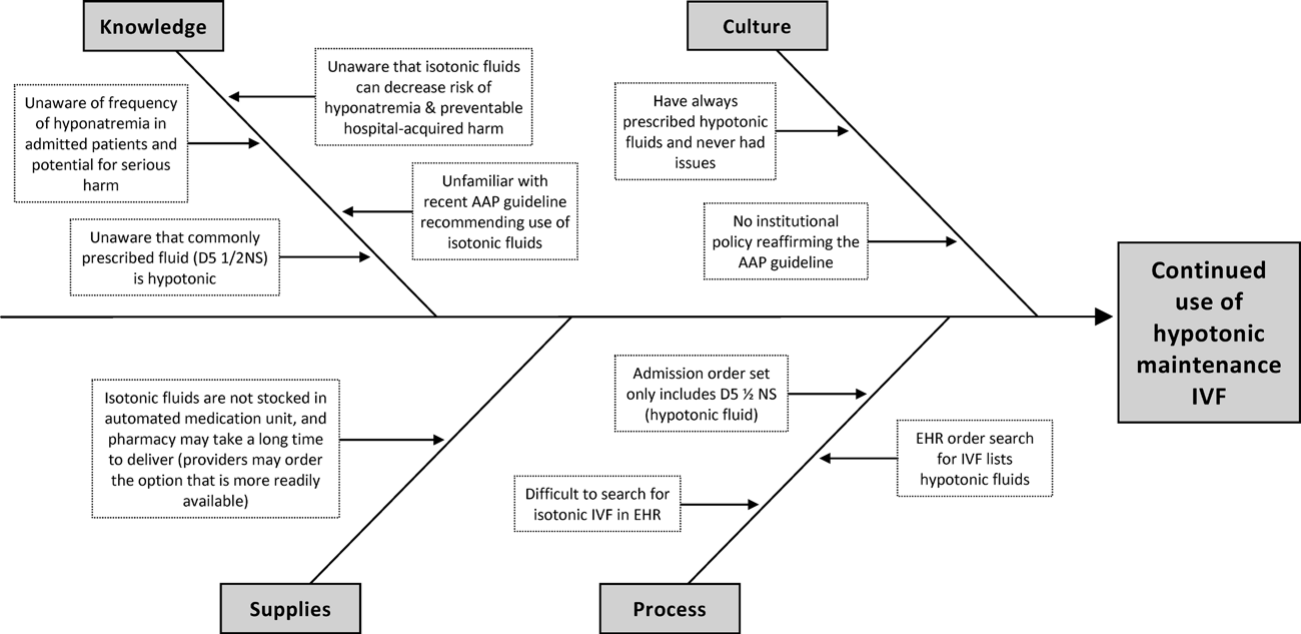
Fishbone diagram. D5 1/2NS, dextrose 5% with ½ normal saline.

A team of physician quality champions at site 1 (S.M., R.M., and L.M.) gathered input from about 15 physicians in their group to identify the root causes of the continued use of hypotonic fluids at their site. Based on this input, the team at site 1 developed a Fishbone diagram (Fig. 1), which revealed that a lack of awareness of the AAP guidelines and a lack of knowledge of the risk and dangers of hyponatremia led to continued use of hypotonic fluids. This was compounded by the difficulty of ordering providers to find the appropriate isotonic fluids orders in the electronic health record (EHR). Furthermore, even when ordered appropriately, the order was often changed as the fluid was not readily available on the unit.

**Fig. 2.**
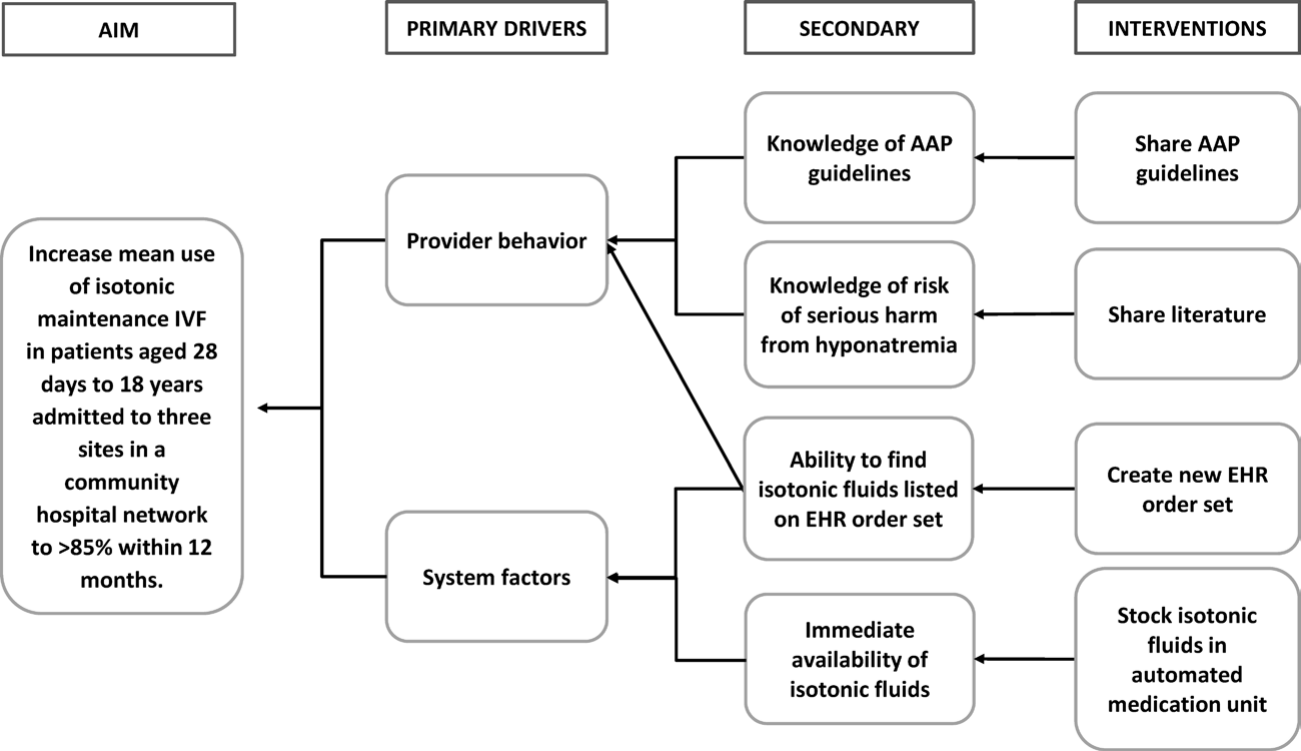
Key driver diagram.

We planned interventions addressing the two primary drivers of provider behavior and systems factors (Fig. 2). Interventions included: (1) education, (2) an IVF order set, and (3) stocking of automated medication units. The quality champions presented the updated AAP guidelines at regular staff meetings, which included physician and nursing groups, and reiterated the guidelines during the orientation of new clinicians. Additionally, physicians and pharmacists collaborated to create an IVF order set in our EHR that would be easy to locate and which encouraged the use of isotonic fluids. The hypotonic fluid choices in the order set were collapsed to make them slightly more difficult to find. The order set also linked to the AAP guidelines and clinical pathway from the associated tertiary care children’s hospital. Previously, a search for orders in the EHR for IVF listed only hypotonic fluids, and the admission order set included hypotonic fluids. Finally, our pharmacy staff ensured automated medication units were adequately stocked with isotonic fluids to address availability. Providers were informed of the new order set and stocking of the automated medication units through a group email. We implemented these interventions at site 1 beginning in October 2019 and expanded to site 2 in February 2020 followed by site 3 in November 2020. These interventions were implemented in a stepwise manner at sites 1 and 2 but in a single instance at site 3.

### Study of the Intervention

We extracted data from the EHR, including patient age at admission, admission date, and type of maintenance fluid used, and established a baseline proportion of average monthly use of isotonic maintenance IVF over at least 10 months for each site. To evaluate the impact of interventions, we compared the proportion of isotonic maintenance fluid orders among all orders for maintenance IVF before and after the introduction of interventions separately for each site. Data collection continued until February 2022 at sites 1 and 3 and until January 2022 at site 2, beyond the initial intended period due to low inpatient volumes during the COVID-19 pandemic. In the analysis, we included patients aged 28 days to 18 years old who were admitted to inpatient pediatric units and received maintenance IVF at a rate of at least 10 mL/hour. We excluded admissions of patients admitted in diabetic ketoacidosis.

### Measures

The main outcome measure was the average monthly proportion of isotonic IVF orders among eligible admissions receiving maintenance IVF. As patients could have multiple fluid orders during an admission, we tracked the proportion of orders for isotonic fluids. We considered D5%–0.9% saline and D5-Lactated Ringer as isotonic maintenance fluids, whereas D5%–0.45% saline and D5%–0.2% saline solutions, each with or without KCl were considered hypotonic. As a balancing measure, we tracked adverse events from hypo- or hypernatremia through institution-specific reporting systems. We monitored fluid status and blood pressure using routine vital signs, input/output monitoring, and physical examination. We performed electrolyte monitoring according to clinical judgment.

### Data Analysis

We used statistical process control charts to analyze the primary outcome measure and applied rules for special cause variation. The standard for determining special cause variation was eight consecutive points above or below the centerline with a theory for the change, for example, a coincident intervention.^14^ Since the ratio of observed to expected variation based on the binomial distribution in each of our samples was greater than the 95% upper confidence limit, consistent with significant overdispersion, there was risk of false alarms in the statistical process control charts. We thus instead used a Laney P (or P′) chart for appropriate control limits.^15^

### Ethical Considerations

This project was undertaken as a QI Initiative and does not constitute human subjects’ research.

## RESULTS

**Fig. 3.**
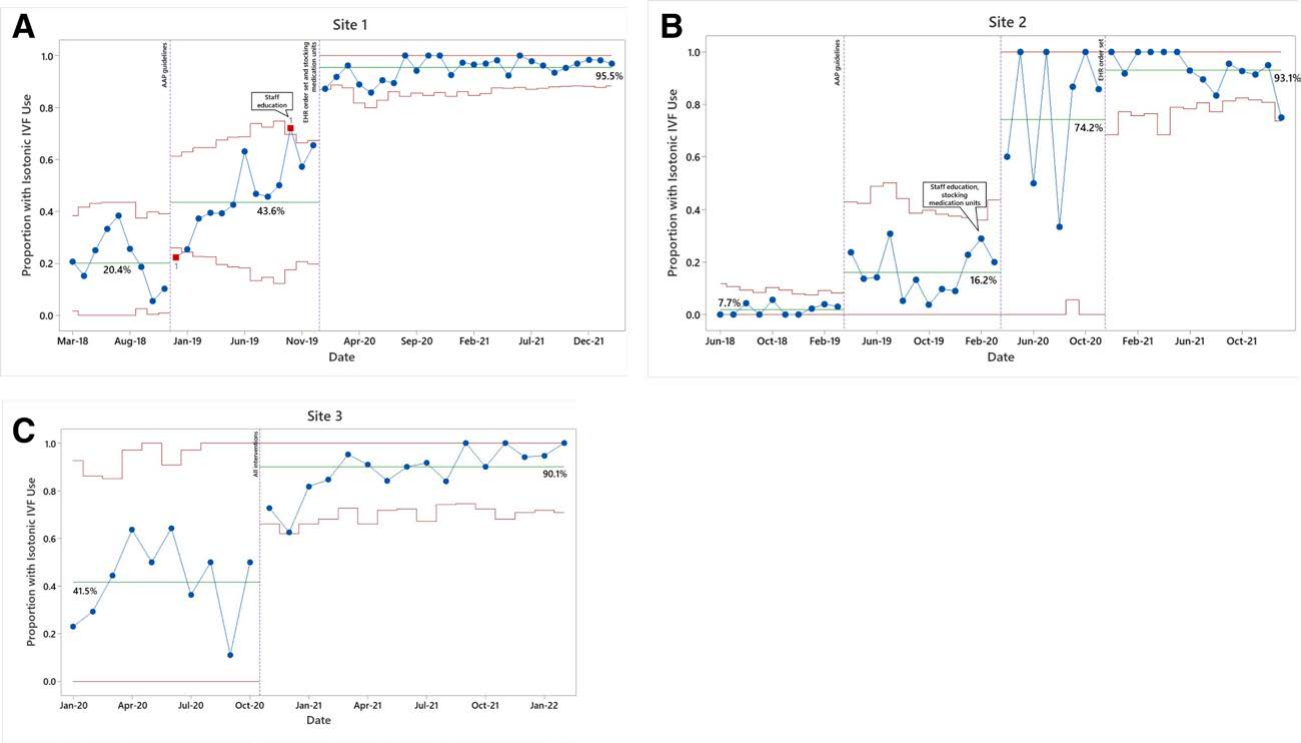
Monthly Proportion of isotonic maintenance fluid orders. A–C, Statistical process control Laney P′ chart by site.

During the preintervention period, we analyzed 825 orders across the three sites and 2,689 orders after interventions were implemented. At site 1, the proportion had increased preintervention from approximately 20% before the publication of the AAP guideline to 44% after guideline publication (Fig. 3A). It rose to approximately 96% after interventions. At site 2, the baseline proportion was approximately 8% and changed slightly after guideline publication. It increased to about 74% after education and stocking the automated dispensing unit and to 93% after the IVF order set (Fig. 3B). The baseline proportion at site 3 after the publication of AAP guidelines was 42% and increased to 90% after implementing interventions (Fig. 3C).

There were no reported adverse events due to hypo- or hypernatremia. We did not have any adverse events which would necessitate follow-up laboratories or prolong length of stay. Routine vital signs, input/output monitoring, and physical examination did not detect any fluid retention or hypertension in our population.

## DISCUSSION

With this QI initiative, we exceeded the goal of increasing the use of isotonic IVF in admissions of patients requiring maintenance fluids aged 28 days to 18 years to >85%, with all three sites achieving an average use of isotonic fluids at or above 90%. Sites 1 and 2 used a stepwise approach, whereas site 3 implemented the project later and, learning from the other sites, introduced all the interventions at once with a more immediate result.

Other recent publications have described the use of targeted education, clinical support in the EHR, and order set revisions as we used, as well as performance practice review in successfully making the transition to isotonic IVF in children in a pediatric emergency department (ED),^16^ at an urban academic tertiary medical center,^17^ on a resident-led inpatient unit,^18^ and as part of a large multicenter AAP-sponsored project.^19^ To our knowledge, this is the first report of IVF guideline implementation in a community hospital setting.

We applied the knowledge gained at our first community site, combined with local knowledge of subsequent sites to adopt the recommendations more efficiently at the third site. Education helped to some degree, but modifying order sets and stocking isotonic fluids on the units helped much more in exceeding our goal. Additionally, having the hypotonic fluid selections collapsed in the order set may approach a level of reliability 2 intervention,^20^ which may have contributed to the success of our efforts. We did not use formal performance review, but the small staff size at our sites meant more informal provider encouragement was possible. We anticipate ease in sustaining our new practice because EHR changes with clinical support and local stocking of isotonic fluids remain a standard at our sites. With this culture shift, we encourage newly hired providers to review the current guidelines and utilize order sets to ensure appropriate IVF use is sustained. A limitation is that we did not routinely monitor electrolyte to detect changes in Na or Cl at any of our sites.

We have shown that we can leverage both our large tertiary care hospital resources, and the power of our experiences and expertise as community hospitalists to successfully adapt our methods of change to our local cultures and individual workflows. As a next step, we hope to expand the use of isotonic maintenance fluids to the local EDs to prevent order changes after admission leading to wastage of hypotonic fluid bags ordered in the ED. We will also consider the use of an automated Best Practice Advisory to reinforce ordering practices in all settings. We demonstrate to others that small and large community hospitals can successfully transition to isotonic fluids as a standard of care, and that a small team committed to improvement at different sites can function well together and support each other as the change is made.

## CONCLUSION

A combination of interventions aimed at provider behavior and systems factors were critical to successful adoption of the AAP guideline regarding the use of maintenance isotonic IVF in hospitalized children in the community hospital setting.

## ACKNOWLEDGMENTS

The authors thank Kristin McNaughton, MHS, for assistance with editing and April Taylor, MHA, MS, for guidance early in the project.
